# Bioefficacy of *Epaltes divaricata* (L.) *n*-Hexane Extracts and Their Major Metabolites against the Lepidopteran Pests *Spodoptera litura* (fab.) and Dengue Mosquito *Aedes aegypti* (Linn.)

**DOI:** 10.3390/molecules26123695

**Published:** 2021-06-17

**Authors:** Kesavan Amala, Sengodan Karthi, Raja Ganesan, Narayanaswamy Radhakrishnan, Kumaraswamy Srinivasan, Abd El-Zaher M. A. Mostafa, Abdullah Ahmed Al-Ghamdi, Jawaher Alkahtani, Mohamed Soliman Elshikh, Sengottayan Senthil-Nathan, Prabhakaran Vasantha-Srinivasan, Patcharin Krutmuang

**Affiliations:** 1 Department of Biotechnology, St. Peter’s Institute of Higher Education and Research, Avadi-600 054 Chennai, Tamil Nadu, India; amalavenkey@gmail.com; 2 Division of Bio-Pesticides and Environmental Toxicology, Sri Paramakalyani Centre for Excellence in Environmental Sciences, Manonmaniam Sundaranar University, Alwarkurichi, 627412 Tirunelveli, Tamil Nadu, India; karthientomology@gmail.com; 3 Department of Internal Medicine, Hallym University College of Medicine, Chuncheon 200 704, Korea; vraja.ganesan@gmail.com; 4 Department of Biochemistry, St. Peter’s Institute of Higher Education and Research, Avadi-600 054 Chennai, Tamil Nadu, India; nrkishnan@gmail.com (N.R.); srikamalesh251@gmail.com (K.S.); 5 Department of Botany and Microbiology, College of Sciences, King Saud University, P.O. Box 22452, Riyadh 11495, Saudi Arabia; amus@ksu.edu.sa (A.E.-Z.M.A.M.); abdalghamdi@ksu.edu.sa (A.A.A.-G.); jsalqahtani@ksu.edu.sa (J.A.); melshikh@ksu.edu.sa (M.S.E.); 6 Department of Entomology and Plant Pathology, Faculty of Agriculture, Chiang Mai, University, Muang, Chiang Mai 50200, Thailand; 7 Innovative Agriculture Research Center, Faculty of Agriculture, Chiang Mai University, Chiang Mai 50200, Thailand

**Keywords:** Epaltes, *n*-HDa, *n*-ODa, lepidopteran, dengue vector, insecticide

## Abstract

The present research investigated the chemical characterization and insecticidal activity of *n*-Hexane extracts of *Epaltes divaricata* (NH-EDx) along with their chief derivatives *n*-Hexadecanoic acid (*n*-HDa) and *n*-Octadecanoic acid (*n*-ODa) against the dengue vector *Aedes aegypti* and lepidopteran pest *Spodoptera litura*. Chemical screening of NH-EDx through GC–MS analysis delivered nine major derivatives, and the maximum peak area percentage was observed in *n*-Hexadecanoic acid (14.63%) followed by *n*-Octadecadienoic acid (6.73%). The larvicidal activity of NH-EDx (1000 ppm), *n*-HDa (5 ppm), and *n*-ODa (5 ppm) against the *A. aegypti* and *S. litura* larvae showed significant mortality rate in a dose-dependent way across all the instars. The larvicidal activity was profound in the *A. aegypti* as compared to the *S. litura* across all the larval instars. The sublethal dosages of NH-EDx (500 ppm), *n*-HDa (2.5 ppm), and *n*-ODa (2.5 ppm) also showed alterations in the larval/pupal durations and adult longevity in both the insect pests. The enzyme activity revealed that the α- and β-carboxylesterase levels were decreased significantly in both the insect pests, whereas the levels of GST and CYP450 uplifted in a dose-dependent manner of NH-EDx, *n*-HDa, and *n*-ODa. Correspondingly, midgut tissues such as the epithelial layer (EL), gut lumen (GL), peritrophic matrix (Pm), and brush border membrane (BBM) were significantly altered in their morphology across both *A. aegypti* and *S. litura* against the NH-EDx and their bioactive metabolites. NH-EDx and their bioactive metabolites *n*-HDa and *n*-ODa showed significant larvicidal, growth retardant, enzyme inhibition, and midgut toxicity effects against two crucial agriculturally and medically challenging insect pest of ecological importance.

## 1. Introduction

Phytochemicals are generally secondary metabolites that aid as a solid defensive action in plants against the constant selection pressure of herbivore predators, microbial pathogens, and other ecological risks [1]. Plant extracts hold surplus phytocompounds that are not utilized as nutrients for their growth [2]. Presently, research on insect toxicology has chiefly focused on green extracts and their major derivative warehouse to manage harmful pests of both agricultural and medical importance. Generally, plant extracts/essential oils/bioactive compounds have performed significant roles as mosquitocides and insecticides specially targeting lepidopteran insects [3,4]. The class of “plant extracts” generally covers a huge blend of phytochemicals, many of which delivered a higher impact in controlling insect pests [5]. Global researchers have proved that plant extracts and their bioactive compounds predominantly control harmful agriculture pests and disease-spreading arthropods with similar toxicity as displayed by chemical pesticides [6,7,8]. Moreover, plant extracts have been widely utilized by human beings across the world and merged into integrated pest management (IPM) for effective control measures [9]. Previous natural product research has evidenced that plant-derived phytocompounds have multipotent toxicity against insect pests, with diverse functions such as insect growth inhibitors, larvicides, repellents, and adulticides, as well as having ovipositional attractants [10,11,12,13]. 

Insect pests play a crucial role in damaging agricultural crops apart from other natural problems. In particular, Lepidoptera, the largest family, embraces most caustic pests that can damage crops both directly and indirectly [14]. Among the lepidopteran pests *Spodoptera litura* Fab. (Lepidoptera: Noctuidae) is most significant and harmful pest, reported to target >150 plant species in south Asian countries [15]. *S. litura* is a firm flier and splits a wide-ranging distance annually all over the summer season. It is recognized as the most harmful agriculture pest of tropical nations such as China, Japan, and India, as it causes significant financial damage to diversified vegetables and grain crops [15,16].

Correspondingly, dengue is the most rampant human arbovirus, infecting half of the global population across 120 countries [17]. The chief culprit for spreading this disease is *Aedes aegypti* (Diptera: Culicidae) Linn., which is considered to be one of the crucial mosquito vectors capable of spreading the dengue virus and causing a significant mortality rate by spreading dengue hemorrhagic fever (DHF) [7]. It is estimated that >2.5 billion people are at high risk of dengue in more than 100 nations [18,19]. In India, it is estimated that 34% of global cases occur, as the disease is endemic, and that it mainly spreads four different serotypes: DENV-1, -2, -3, and -4 [20]. Synthetic chemical application is the major tool in managing both pests (*S. litura* and *A. aegypti*). However, the selection pressure levied by commercial insecticides is uplifting the resistance ratio in both of these pests and more importantly causing adverse effects to nontargets [21,22,23]. Green-based products such as crude extracts, essential oils, and chief derivatives have been consider as a potential insect pest management agent.

Chemical pesticides are recognized as chief agents to manage insect pests due to their general acceptance [24]. Resistance to major commercial insecticides has been associated with an upsurge in the level of metabolism in insects, such as the up-regulation of detoxifying enzymes or enzyme structural changes that increase the capacity of metabolic rate [25,26,27,28,29]. Previous research on diverse plant extracts and chief metabolites against mosquito vectors or agriculture pests has delivered that many botanical sources proved as potential alternatives, or as rotational chemistries, to heavily-used synthetic chemical insecticides [30]. Plant derivatives may deliver alternative sources of commercial pesticides, since they are enriched with bioactive insecticidal compounds that are easily degradable [2,31,32]. 

The genus *Epaltes* is widely used by traditional healers in South Asian countries such as Sri Lanka, India, Java, and China for treating diverse medical complications including jaundice, acute dyspepsia, and urethral discharges [33]. Traditionally, the roots of *Epaltes* species are utilized as healing tonics for treating insect or reptile bites [34]. Among these species, *Epaltes divaricata* (L.), which belongs to the family Compositae, is a traditional medicinal plant used in Ayurveda medicine for healing diverse medical problems [35,36]. Previous research on ethyl acetate extracts of *E. divaricata* displayed profound inhibition against *Staphylococcus aureus* multiple drug resistant strains (MRSA) [37]. However, the chemical characterization of these extracts in screening their phytochemicals and their insecticidal activity remains scanty. 

Thus, the present research aimed to investigate the following objectives: (a) chemical characterization and larvicidal activity of *n*-Hexane extracts of *E. divaricata* (NH-EDx), along with their chief derivatives *n*-Hexadecanoic acid (*n*-HDa) and *n*-Octadecanoic acid (*n*-ODa), against *A. aegypti* and *S. litura*; (b) detection of developmental changes in the larval and pupal duration in both insect pests; (c) detection of enzyme inhibition activity of NH-EDx, *n*-HDa, and *n*-ODa in both lepidopteran pest and dengue vector and (d) evaluation of gut-histological activity in both of the pests after treatment with the extracts and major derivatives of *E. divaricata*.

## 2. Methodology

### 2.1. Insect Culture

*A. aegypti* rearing was maintained unexposed to any chemicals at the Insect Toxicology Laboratory (ITL) at St. Peter’s Institute of Higher Education and Research, Chennai, India, and the complete rearing procedure followed the adapted methodology of Thanigaivel et al. [19]. The cultures were conserved, and all the experiments were carried out in our laboratory at 27 ± 2 °C and 75–85% RH under a 14:10 L/D photoperiod without exposure to any chemical insecticides. Brewer‘s yeast, dog biscuits (Choo Stix Biskies), and algae collected from pools in a ratio of 3:2 were fed as a diet to the larvae. Pupae that emerged from the larvae were shifted to a plastic cup (round, 250 mL capacity) containing tap water and located in breeding cages (60 × 60 × 60 cm dimensions) for adult emergence. Wet raisins (dried grapes) and 10% sucrose solution soaked in cotton were fed to the adults. The adult females were destitute of sucrose from 6h and then provided a mouse placed in a breeding cage overnight for blood feeding. Adult mosquitoes were maintained under sterile conditions as similar to the larvae. The first-generation larvae were used for conducting the experiment.

Correspondingly, the *S. litura* culture was maintained since 2019 without exposure to any pesticides or chemicals under in vitro conditions. The eggs were sterilized in surface using 10% formaldehyde for 10 min and washed with double distilled water for 5 min, then finally air-dried and allowed to hatch. The adults that emerged were shifted to a hygienic plastic container having castor leaves for oviposition and were fed with 8% honey solution on cotton wool located in a small glass cap. Generally, eggs laid in batches on paper were kept for hatching in sterilized plastic boxes. The environment was set as 25–27 °C and 60–70% relative humidity in an ecologically controlled chamber. The day/night period was adjusted to 13:11 h. Second-generation larval instars were used for the bioassay.

### 2.2. Plant Harvesting and Crude Extract Preparation

**Figure 1:**
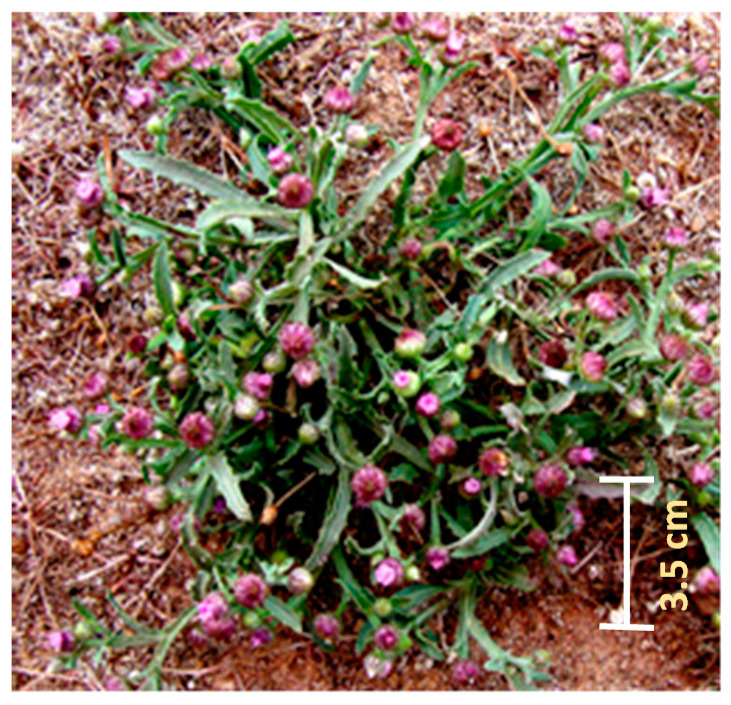
External morphology of *Epaltes divaricata* (L.) Cass. collected from the southwestern Ghats region of Tirunelveli, Tamil Nadu, India.

Fresh leaves of the plant material *Epaltes divaricata* (L.) Cass. were harvested during the early morning hours (6.00 AM) from the Southern Ghats region, Tirunelveli, Tamil Nadu, India. Harvesting was authenticated by a botanist from Plant Biology and Plant Biotechnology, Presidency College, Chennai, India (Voucher Specimen No.00629), and leaves were deposited in the Department of Botany, Captain Srinivasa Murti Drug Research Institute for Ayurveda, Chennai, India for reference. The external morphology of *E. divaricata* is displayed in Figure 1.

The collected plant material was shade-dried under laboratory conditions (27 ± 2 °C). The dried leaves were powdered finely using a blender, and the dried leaf powder was extracted by a a Soxhlet apparatus using *n*-hexane solvent as adapted from Ponsankar et al. [11]; Further, 500 g air-dried plant powder was dissolved with 700 mL of *n*-hexane (60–80 °C) and then extracted with 750 mL distilled *n*-hexane in a Soxhlet apparatus for 16 h. The extract was filtered and concentrated in a rotary flash evaporator at 60 °C; the concentrated *n*-hexane extract was poured into the same volume of methanol while stirring and then filtered. The solvent was further removed by vacuum evaporation in a rotary evaporator, and the deposits were stored at 4 °C in an airtight glass bottle for further experiments. The yield of *n*-hexane extract was 2.03 g.

### 2.3. Characterization of Plant Volatile through GC–MS

Two microliters of NH-EDx were dissolved in methanol (HPLC-grade) and projected to GC and MS JEOL GC mate prepared with secondary electron multiplier (JEOL GCMATE II GC-MS (Agilent Technologies 6890N Network GC system)). The HP5 column was linked with silica 55 m × 0.25 mm I.D. Analysis conditions were (i) HPLC-grade—20 min at 100 °C; (ii) column temperature—3 min at 235 °C; and (iii) injector temperature 240 °C. Helium was the carrier gas, and the split ratio was 5:4. Further experimental procedure was adapted from the previous methodology [37]. The molecular structure, molecular weight, and formula of the individual compounds of test materials were ascertained by interpretation of the mass spectrum from GC–MS using the database of the National Institute of Standards and Technology (NIST).

### 2.4. Chemicals

GC–MS analysis results of NH-EDx delivered peak areas in *n*-Hexadecanoic acid (*n*-HDa) followed by *n*-Octadecadienoic acid (*n*-ODa). Thus, to detect the efficacy of these selective metabolites of NH-EDx against insect pests, the plant chemicals were commercially purchased from Sigma-Aldrich (≥99% (capillary GC)).

### 2.5. Larvicidal Bioassay

#### 2.5.1. *A. aegypti* Larvicidal Assay

The second, third, and fourth instar larvae were placed into 200 mL sterile disposable plastic cups each covering 20 mL of different dosages of NH-EDx (200, 400, 600, 800, and 1000 ppm) and the bioactive derivatives *n*-HDa and *n*-ODa (1, 2, 3, 4, and 5 ppm) and incubated at 27 °C. A solution containing *n*-Hexane (0.1%) was employed as the negative control. During the treatment period, larval food was added to each test cup, particularly if high mortality was noted in the control. Larvae were considered dead when they were unable to reach the surface of the solution when the cups were disturbed. The larvae were considered moribund if, at the end of 24 h, they showed no sign of swimming movements even after gentle touching with a glass rod. The dead and moribund larvae were recorded after 24 h of treatment. Larvicidal assay was adapted from the World Health Organization [38].

#### 2.5.2. *S. litura* Larvicidal Assay

Larvicidal assays of NH-EDx and their derivatives *n*-HDa and *n*-ODa were executed against the II, III, and IV instars of *S. litura* larvae with discriminating dosages utilized in the *A. aegypti* larvicidal assay (Section 2.5.1). The treatment dosages were gushed in sterile castor leaves and allowed to air dry. Hexane (0.1%) was used alone as a control. Second to fourth instars larvae were starved for 4 h were exposed to the treated leaves and kept under fixed conditions (28 ± 2 °C and 75% relative humidity). The rate of mortality was observed every 24 h, compared with the control, and recorded. The calculated mortality data percentage was examined using Probit analysis to determine the lethal concentrations (LC_50_ and LC_90_). The treatments were replicated five times, and each replicated set contained one control. Analyses of the follow-up assay were carried out according to the Probit method. Percentage mortality (1) was calculated by using Abbott’s formula [39] (2).

(1) $$\mathrm{Percentage}\;\mathrm{of}\;\mathrm{mortality}=\frac{\mathrm{Number}\;\mathrm{of}\;\mathrm{dead}\;\mathrm{larvae}}{\mathrm{Number}\;\mathrm{of}\;\mathrm{larvae}\;\mathrm{introduced}}\times 100$$

(2) $$\mathrm{Corrected}\;\mathrm{percentage}\;\mathrm{of}\;\mathrm{mortality}=(1-\frac{n\;\mathrm{in}\;T\;\mathrm{after}\;\mathrm{treatment}}{n\;\mathrm{in}\;C\;\mathrm{after}\;\mathrm{treatment}})\times 100$$

### 2.6. Larval and Pupal Duration Assay

The effect of sublethal dosages (LC_50_—dosage responsible to cause 50% insect mortality in a single exposure) of plant extracts (NH-EDx) along with their major phytochemicals (*n*-HDa and *n*-ODa) against the larval and pupal stages of both *A. aegypti* and *S. litura*, with discriminating sublethal dosages (NH-EDx—100, 200, 300, 400, and 500 ppm; *n*-HDa and *n*-ODa—0.5, 1.0, 1.5, and 2.0 ppm) were prepared. For analyzing *A. aegypti* larval and pupal durations, the sublethal dosages were subjected in an enamel tray of 30 × 25 × 5 cm dimensions. Fifty eggs were released into enamel tray in treated water and allowed to hatch. For analyzing *S. litura* larval and pupal duration, the sublethal dosages were dipped in sterile castor leaves, the eggs were released, and their duration to become larvae and pupae in days were calculated and compared with the control, which was treated with hexane alone. The total larval duration (days) was calculated from hatching to pupation. Pupae were placed in a small container closed with a transparent mesh. The pupal duration (days) was calculated from the pupal molt to adult emergence.

### 2.7. Enzyme Assays

#### 2.7.1. α- and β-Carboxylesterase Activity

The enzyme activity of α- and β-carboxylesterase in the *A. aegypti* and *S. litura* was determined using the adapted methodology of Agra-Neto et al. [40], The enzyme activity was investigated by the expression of one unit of active enzyme, which was determined as the amount of enzyme essential to generate 1 µmol of α- or β-naphthol expressed per minute.

#### 2.7.2. GST and CYP450 Activity

Glutathione-S transferase activity (GST) was expressed as µmol/mg protein/min substrate conjugated, and cytochrome P450 activity (CYP450) was expressed as l mol 7-OH/mg larvae/min. The complete protocol was followed based on the GST assay kit (Sigma-Aldrich (Catalog 0410, Bangalore)) that was used to evaluate the conjugation of the thiol group of glutathione to the 1-chloro-2, 4- dinotrobenzene (CDNB) substrate. CYP450 activity was determined based on the measurement of ethoxycoumarin-*O*-de-ethylase level in the body walls as adapted from Thanigaivel et al. [41].

### 2.8. Gut-Histological Assay

The NH-EDx-, *n*-HDa-, *n*-ODa-, treated and control- fourth instars of *A. aegypti* and *S. litura* were fixed in Bouin’s solution overnight, flowingly dehydrated, and mounted in wax blocks using paraffin. Larval blocks of tissue were segmented using the microtome instrument (Leica, Germany), and the active sections were mounted on sterile microscopic glass slides, stained with eosin and hematoxylin, and observed for histopathological changes and photographed using light a microscope (Optika vision lite 2.0 ML) pre-connected with a laptop. Midgut cells of *A. aegypti* and *S. litura* were photographed, and the changes in the midgut cells of treated larvae were observed and matched with control [6].

### 2.9. Data Analysis

The larvicidal experimental data were exposed to analysis of variance (ANOVA and square root transformed percentages), and the obtained statistical results were considered as a five replicate means. Statistical significance within each larvicidal group was analyzed using Tukey’s multiple range test (significance at *p* < 0.05), and the lethal concentrations of larvae in 24 h were calculated by Probit analysis with a dependability interval of 95% using the Minitab^®^ 17 program. For determining enzyme inhibition level, Microcal Software (Sigma plot 11) was used to plot the graphs.

## 3. Results

### 3.1. Chemical Characterization of NH-EDx

Chemical screening of NH-EDx through GC–MS analysis showed nine major derivatives, and the maximum peak area percentage was observed in *n*-Hexadecanoic acid (14.63%) followed by *n*-Octadecadienoic acid (6.73%). Correspondingly, the NH-EDx was also composed of Hexacosane (6.13%), *n*-Hexatriacontane (5.13%), Ethyl Hexadecanoate (1.32%), Tetratriacontane (1.3%), *n*-Eicosane (1.13%), Neophytadiene (0.83%), and finally Hexadecane (0.75%) (Table 1). All of these derivatives might play a significant role in causing mortality rate in both of the pests. Since these metabolites are the major active derivatives of the NH-EDx, they all synergistically act together, which results in displaying profound activity against both the pests. Despite this, the major peak area derivatives *n*-HDa followed by *n*-ODa might signify a profound level of contribution against the insect pests. Thus, these two compounds were purchased commercially, and their bioefficacy against both the pests (*A. aegypti* and *S. litura*) was examined parallel to that of the NH-EDx.

### 3.2. Larvicidal Activity

**Figure 2:**
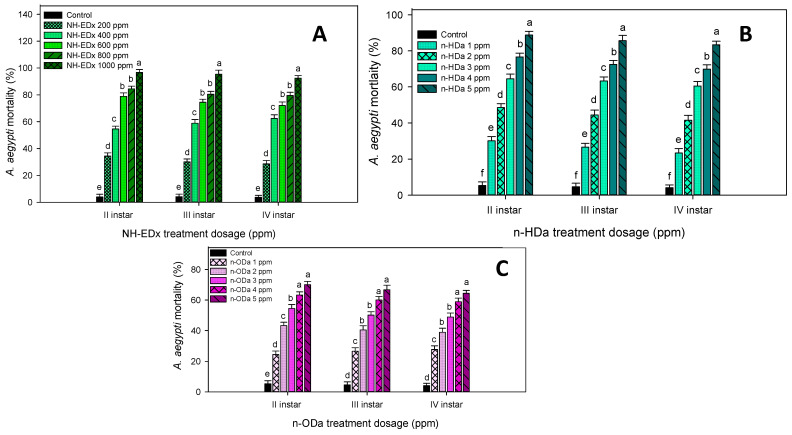
Percentage mortality of second, third, and fourth instar larvae of *A. aegypti* after treatment with NH-EDx (**A**), *n*-HDa (**B**), and *n*-ODa (**C**). Means (±SEM) followed by the same letters above bars indicate no significant difference (*p* ≤ 0.05) according to a Tukey’s test.

The larvicidal activity of NH-EDx against *A. aegypti* larvae showed a significant mortality rate in a dose-dependent way across all the instars. The mortality rate was profound at the maximum dosage (NH-EDx—1000 ppm), and it was significantly different from other dosages in II instars (97.65%—F_4,20_ = 28.68, *p* ≤ 0.001), III instars (94.11%—F_4,20_ = 20.32, *p* ≤ 0.001), and IV instars (92.94%—F_4,20_ = 18.88, *p* ≤ 0.001). Despite this, there was no significant difference between NH-EDx 800 and 600 ppm across all the treated larval instars. Even so, all of the treatment dosages were significantly different from control (Figure 2A).

Similar results were obtained from phytochemical treatment (*n*-HDa and *n*-ODa). The mortality rate was found to be concentration-dependent. There was a significant difference in the mortality rate at the maximum dosage of 5 ppm in II instars (98.86%—F_4,20_ = 14.22, *p* ≤ 0.001), III instars (95.21%—F_4,20_ = 14.55, *p* ≤ 0.001), and IV instars (93.89%—F_4,20_ = 19.18, *p* ≤ 0.001), in the *n*-HDa treatment (Figure 2B). Likewise, a higher mortality rate was recorded in the 5 ppm dosage of *n*-ODa treatment in II instars (98.93%—F_4,20_ = 13.33, *p* ≤ 0.001), III instars (96.87%—F_4,20_ = 17.89, *p* ≤ 0.001) and IV instars (94.75%—F_4,20_ = 16.90, *p* ≤ 0.001) (Figure 2C). However, there was no significant difference between *n*-HDa or *n*-ODa treatment.

**Figure 3:**
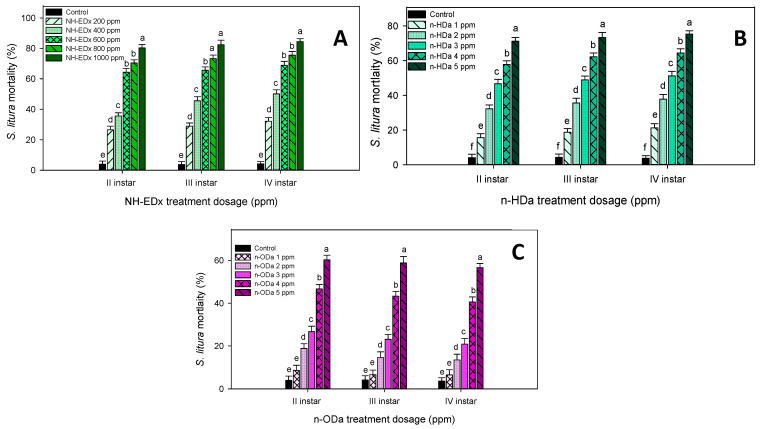
Percentage mortality of second, third, and fourth instar larvae of *S. litura* after treatment with NH-EDx (**A**), *n*-HDa (**B**), and *n*-ODa (**C**). Means (±SEM) followed by the same letters above bars indicate no significant difference (*p* ≤ 0.05) according to a Tukey’s test.

The larvicidal activity of NH-EDx against the lepidopteran pest *S. litura* showed a prominent mortality rate at the maximum lethal dosage of 1000 ppm across the II instars (91.35%—F_4,20_ = 43.84, *p* ≤ 0.001), III instars (89.88%—F_4,20_ = 18.45, *p* ≤ 0.001), and IV instars (86.18%—F_4,20_ = 34.67, *p* ≤ 0.001), and it was significantly different from other treatment dosages and from control (Figure 3A). Like the NH-EDx treatment, the bioactive metabolite *n*-HDa showed a higher mortality rate at the maximum dosage of 5 ppm in II instars (89.45%—F_4,20_ = 17.34, *p* ≤ 0.001), III instars (86.66%—F_4,20_ = 42.15, *p* ≤ 0.001) and IV instars (85.17%—F_4,20_ = 19.45, *p* ≤ 0.001) (Figure 3B). The maximum dosage of *n*-ODa also showed higher larval mortality at the maximum dosage of 5 ppm across II instars (75.45%—F_4,20_ = 18.58, *p* ≤ 0.001), III instars (67.66%—F_4,20_ = 32.90, *p* ≤ 0.001), and IV instars (63.15%—F_4,20_ = 12.45, *p* ≤ 0.001) (Figure 3C).

Overall, the larval mortality rate was found to be higher in the *A. aegypti* than in the *S. litura* across all the dosages; the maximum significance was observed in the 1000 ppm dosage (F_4,20_ = 15.33, *p* ≤ 0.001). Also, the mortality rate of phytocompounds seems to be profound against the *A. aegypti* larvae as compared to the NH-EDx. In contrast, the larval mortality was significant in the crude extract (NH-EDx) as compared to the phytocompounds (*n*-HDa and *n*-ODa) against *S. litura* larvae. The LC_50_ (lethal concentration to kill 50% of larvae) was observed at 500 ppm in the NH-EDx and 2.5 ppm in the *n*-HDa and *n*-ODa across both pests.

### 3.3. Developmental Changes

**Figure 4:**
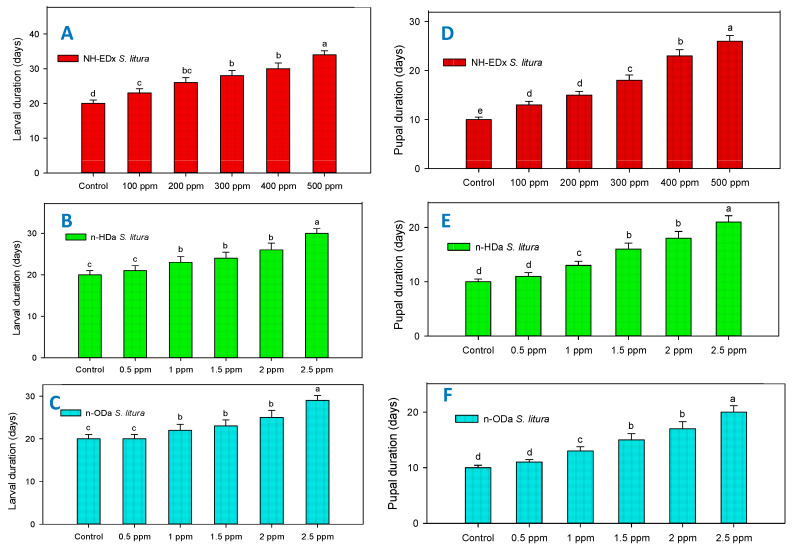
Developmental time in days for *S. litura* larvae after treatment with NH-EDx (**A**), *n*-HDa (**B**), and *n*-ODa (**C**), and for *S. litura* pupae after treatment with NH-EDX (**D**), *n*-HDa (**E**), and *n*-ODa (**F**), respectively. Means (±SEM) followed by the same letters above bars indicate no significant difference (*p* ≤ 0.05) according to a Tukey’s test.

Parallel to larval mortality, the sublethal dosages of NH-EDx (500 ppm), *n*-HDa (2.5 ppm), and *n*-ODa (2.5 ppm) also significantly decreased the growth rates of larvae and pupae of both *A. aegypti* and *S. litura*. The larval and pupal duration of *S. litura* after treatment with NH-EDx (500 ppm) increased significantly as compared to other treatment concentrations and control (larvae: F_4,20_ = 11.33, *p* ≤ 0.001; pupae: F_4,20_ = 17.90, *p* ≤ 0.001) (Figure 4A,D). Correspondingly, the *n*-HDa sublethal dosage also showed significant upsurge in the larval and pupal duration of *S. litura* at the maximum dosage of 2.5 ppm in both larvae (F_4,20_ = 9.13, *p* ≤ 0.001) and pupae (F_4,20_ = 10.63, *p* ≤ 0.001) (Figure 4B,E). Similar results were observed in the *n*-ODa treatment, as the larval and pupal duration of *S. litura* extended prominently when the treatment dosage was increased. However, there was no significant difference in the larval and pupal growth rates between the control and the 0.5 ppm dosage (F_4,20_ = 8.13, *p* ≤ 0.001) (Figure 4C,F).

**Figure 5:**
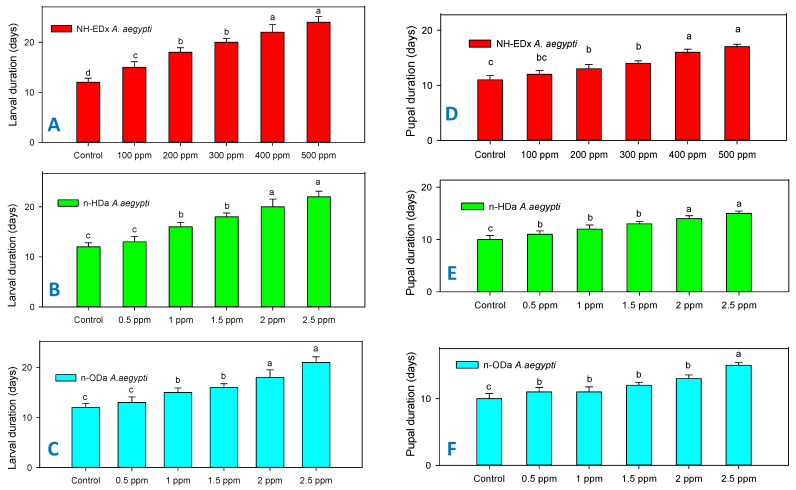
Developmental time in days for *A. aegypti* larvae after treatment with NH-EDx (**A**), *n*-HDa (**B**) and *n*-ODa (**C**) and for *A. aegypti* pupae after treatment with NH-EDx (**D**), *n*-HDa (**E**), and *n*-ODa (**F**), respectively. Means (±SEM) followed by the same letters above bars indicate no significant difference (*p* ≤ 0.05) according to a Tukey’s test.

The larval and pupal duration of *A. aegypti* significantly increased when the treatment dosage of NH-EDx, *n*-HDa and *n*-ODa was increased. The duration of both larvae and pupae was profound in the maximum sublethal dosage of 500 ppm of NH-EDx (larvae: F_4,20_ = 28.94, *p* ≤ 0.001; pupae—F_4,20_ = 14.88, *p* ≤ 0.001), and it was significant compared with other treatment dosages and the control (Figure 5A,D). Similar trends were observed in the *n*-HDa treatment, as the maximum sublethal dosage (2.5 ppm and 2.0 ppm) increased the larval and pupal days of *A. aegypti* steadily as compared to other treatment dosages (Figure 5 B,E). However, there was no significant difference in the duration of larvae and pupae in *A. aegypti* from 0.5 to 1.5 ppm dosages of *n*-ODa (larvae—F_4,20_ = 8.54, *p* ≤ 0.001; pupae—F_4,20_ = 7.32, *p* ≤ 0.001) (Figure 5C,F).

**Figure 6:**
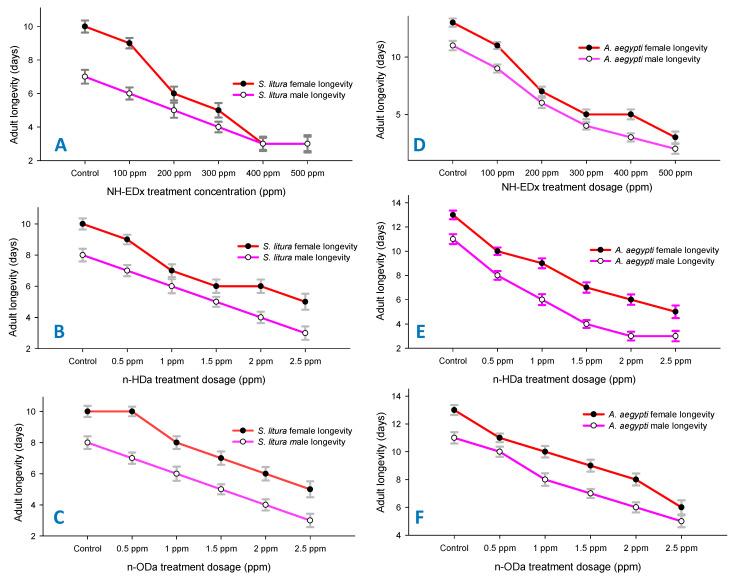
Developmental time in days for *S. litura* adults after treatment with (**A**) NH-EDx, (**B**) *n*-HDa, and (**C**) *n*-ODa, and for *A. aegypti* adults after treatment with (**D**) NH-EDx, (**E**) *n*-HDa, and (**F**) *n*-ODa, respectively.

Correspondingly, the effect on adult longevity (male and female) was also found to be profound in *S. litura* larvae treated with 500 ppm NH-EDx (F_4,20_ = 18.32, *p* ≤ 0.001) (Figure 6A), 2.5 ppm *n*-HDa (F_4,20_ = 13.45, *p* ≤ 0.001) (Figure 6B) and 2.5 ppm *n*-ODa (F_4,20_ = 15.77, *p* ≤ 0.001) (Figure 6C). Likewise, the longevity of adults in *A. aegypti* displayed a significant decline in a dose-dependent manner. Both male and female longevity significantly decreased in 500 ppm NH-EDx (F_4,20_ = 14.88, *p* ≤ 0.001) (Figure 6D), 2.5 ppm *n*-HDa (F_4,20_ = 13.56, *p* ≤ 0.001) (Figure 6E), and 2.5 ppm *n*-ODa (F_4,20_ = 17.11, *p* ≤ 0.001) (Figure 6F).

### 3.4. Enzyme Activity

#### 3.4.1. α-Carboxylesterase

**Figure 7:**
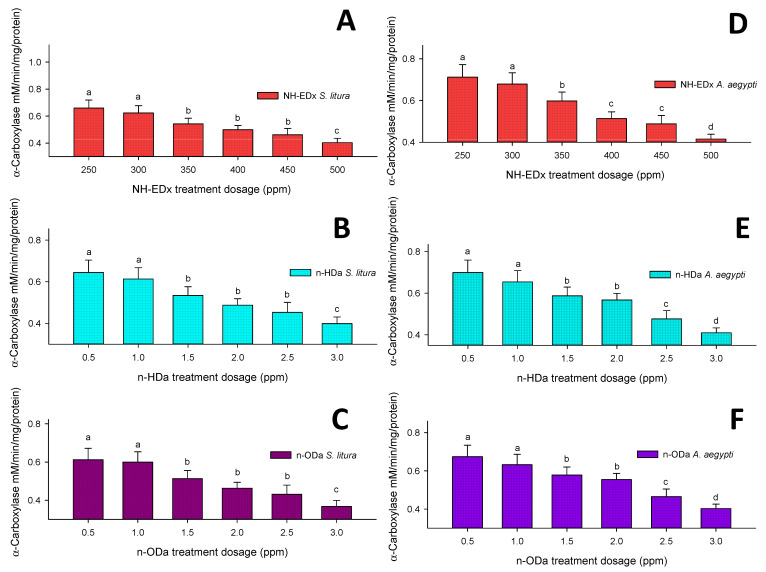
α-Carboxylesterase enzyme activity of the fourth instars of *S. litura* after treatment with (**A**) NH-EDx, (**B**) *n*-HDa, and (**C**) *n*-ODa, and of fourth instars of *A. aegypti* after treatment with (**D**) NH-EDx, (**E**) *n*-HDa, and (**F**) *n*-ODa, respectively. The data were fitted on a polynomial (regression) model. Vertical bars followed by letters indicate standard error (±SEM).

The sublethal dosage of NH-EDx significantly controlled the level of α-carboxylesterase activity in *S. litura* fourth instar larvae in a concentration-dependent manner. The level of α-carboxylesterase reduced at the maximum dosage of 500 ppm (F_4,20_ = 13.55, *p* ≤ 0.001), which result was significant against other treatment dosages and control (Figure 7A). Likewise, the maximum dosages of *n*-HDa and *n*-ODa (3.0 ppm) also exhibited strong inhibition in the level of α-carboxylesterase as compared to other treatment dosages and control (F_4,20_ = 19.77, *p* ≤ 0.001; and F_4,20_ = 23.11, *p* ≤ 0.001, respectively) (Figure 7 B&C). The maximum sublethal dosage of NH-EDx (500 ppm) also reduced the level of α-carboxylesterase in *A. aegypti* fourth instar larvae significantly as compared to other treatment concentrations. However, there was no significant difference between control and 300 ppm NH-EDx (F_4,20_ = 18.22, *p* ≤ 0.001) (Figure 7D). The prominent sublethal dosage of *n*-HDa (3.0 ppm) significantly decreased the enzyme rate as compared to other treatment concentrations and control (F_4,20_ = 19.44, *p* ≤ 0.001) (Figure 7E). Similar trends were observed in *n*-ODa treatment (3.0 ppm), which reduced the α-carboxylesterase in the fourth instars of *A. aegypti* larvae. However, there was no significant difference between the control and the 1.0 ppm dosage (F_4,20_ = 23.22, *p* ≤ 0.001) (Figure 7F).

#### 3.4.2. β-Carboxylesterase

**Figure 8:**
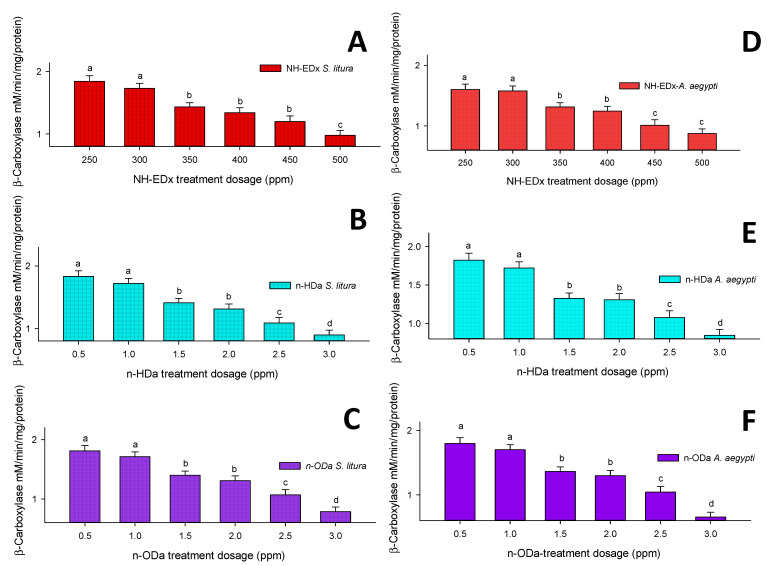
β-Carboxylesterase enzyme activity of the fourth instars of *S. litura* after treatment with (**A**) NH-EDx, (**B**) *n*-HDa, and (**C**) *n*-ODa and of fourth instars of *A. aegypti* after treatment with (**D**) NH-EDx, (**E**) *n*-HDa, and (**F**) *n*-ODa, respectively. The data were fitted on a polynomial (regression) model. Vertical bars followed by letters indicate standard error (±SEM).

The level of β-carboxylesterase in the fourth instars of *S. litura* was significantly reduced by treatment with sublethal dosages of NH-EDx, *n*-HDa, and *n*-ODa, with maximum reductions at 500 ppm (F_4,20_ = 17.47, *p* ≤ 0.001) (Figure 8A), 3.0 ppm (F_4,20_ = 13.99, *p* ≤ 0.001) (Figure 8B), and 3.0 ppm (F_4,20_ = 15.88, *p* ≤ 0.001) (Figure 8C), respectively. Correspondingly, the maximum sublethal dosages of NH-EDx, *n*-HDa, and *n*-ODa decreased the β-carboxylesterase level in the fourth instars of *A. aegypti* significantly at 500 ppm (F_4,20_ = 13.33, *p* ≤ 0.001) (Figure 8D), 3.0 ppm (F_4,20_ = 12.23, *p* ≤ 0.001) (Figure 8E), and 3.0 ppm (F_4,20_ = 16.79, *p* ≤ 0.001) (Figure 8F), respectively.

#### 3.4.3. Glutathione S-Transferase (GST)

**Figure 9:**
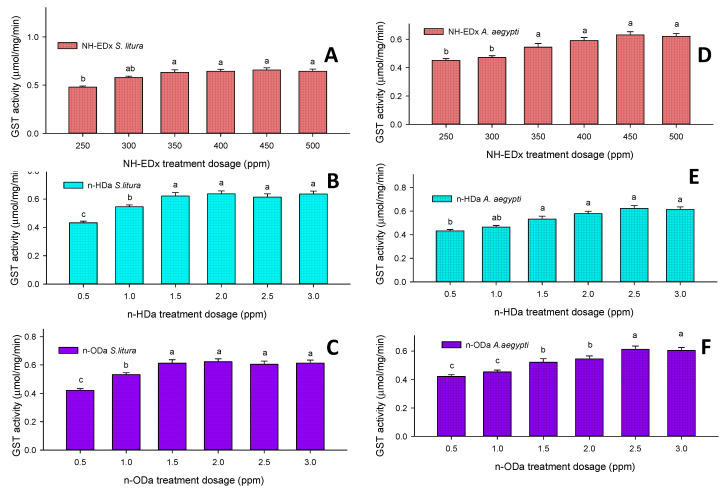
GST enzyme activity of the fourth instars of *S. litura* after treatment with (**A**) NH-EDx, (**B**) *n*-HDa, and (**C**) *n*-ODa and of fourth instars of *A. aegypti* after treatment with (**D**) NH-EDx, (**E**) *n*-HDa, and (**F**) *n*-ODa, respectively. The data were fitted on a polynomial (regression) model. Vertical bars followed by letters indicate standard error (±SEM).

The level of GST increased in a dose-dependent manner in the fourth instars of *S. litura* after treatment with 500 ppm NH-EDx (F_4,20_ = 18.45, *p* ≤ 0.001) (Figure 9A), 3.0 ppm *n*-HDa (F_4,20_ = 21.56, *p* ≤ 0.001) (Figure 9B) and 3.0 ppm *n*-ODa (F_4,20_ = 18.23, *p* ≤ 0.001) (Figure 9C). Likewise, the level of GST increased in a dose-dependent manner in the fourth instars of *A. aegypti* after treatment with 500 ppm NH-EDx (F_4,20_ = 12.35, *p* ≤ 0.001) (Figure 9D), 3.0 ppm *n*-HDa (F_4,20_ = 11.26, *p* ≤ 0.001) (Figure 9E), and 3.0 ppm *n*-ODa (F_4,20_ = 28.33, *p* ≤ 0.001) (Figure 9F). However, there is no significant difference between 500, 400, and 450 ppm doses of NH-EDx or between 3.0, 2.5, and 2.0 ppm doses of *n*-HDa.

#### 3.4.4. Cytochrome P-450 (CYP450)

**Figure 10:**
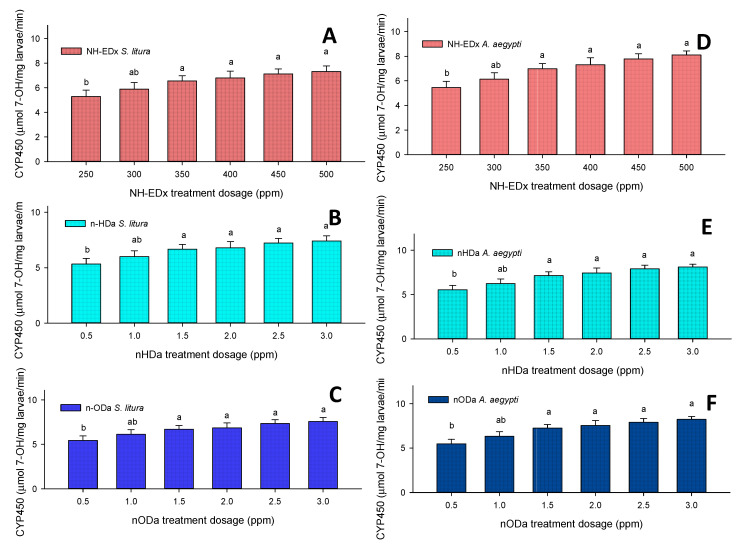
CYP450 enzyme activity of the fourth instars of *S. litura* after treatment with (**A**) NH-EDx, (**B**) *n*-HDa, and (**C**) *n*-ODa and of fourth instars of *A. aegypti* after treatment with (**D**) NH-EDx, (**E**) *n*-HDa, and (**F**) *n*-ODa, respectively. The data were fitted on a polynomial (regression) model. Vertical bars followed by letters indicate standard error (±SEM).

The level of CYP450 was directly proportional to the dosage of the treatment, similarly to GST, in the fourth instars of *S. litura* after treatment with 500 ppm NH-EDx (F_4,20_ = 15.54, *p* ≤ 0.001) (Figure 10A), 3.0 ppm *n*-HDa (F_4,20_ = 18.65, *p* ≤ 0.001) (Figure 10B), and 3.0 ppm *n*-ODa (F_4,20_ = 12.22, *p* ≤ 0.001) (Figure 10C). Likewise, the level of CYP450 increased in a dose-dependent manner in the fourth instars of *A. aegypti* after treatment with 500 ppm NH-EDx (F_4,20_ = 18.45, *p* ≤ 0.001) (Figure 10D), 3.0 ppm *n*-HDa (F_4,20_ = 21.56, *p* ≤ 0.001) (Figure 10E), and 3.0 ppm *n*-ODa (F_4,20_ = 18.23, *p* ≤ 0.001) (Figure 10F). However, there was no significance between the NH-EDx doses of 350, 400, 450, and 500 ppm in either *S. litura* (F_4,20_ = 15.23, *p* ≤ 0.001) or *A. aegypti* (F_4,20_ = 11.88, *p* ≤ 0.001).

### 3.5. Gut-Histological Activity

**Figure 11:**
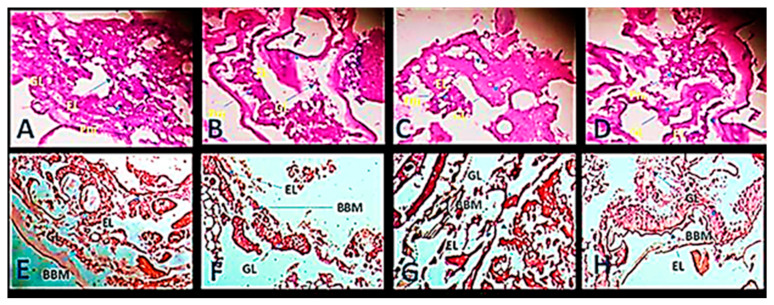
Gut-histological analysis of cross-section of the midgut of fourth instar larvae of *A. aegypti* after treatment with (**A**) control, (**B**) NH-EDx (500 ppm), (**C**) *n*-HDa (5 ppm), and (**D**) *n*-ODa (5 ppm) and of fourth instar larvae of *S. litura* after treatment with (**E**) control, (**F**) NH-EDx (500 ppm), (**G**) *n*-HDa (5 ppm), and (**H**) *n*-ODa (5 ppm). EL: epithelial layer; GL: gut lumen; Pm: peritrophic matrix; BBM: brush border membrane.

The midgut cells were severely infected in the fourth instar larvae of *A. aegypti* after treatment with sublethal dosages of NH-EDx (500 ppm) (Figure 11B), *n*-HDa (Figure 11C), and *n*-ODa (Figure 11D) as compared to the control (Figure 11A). The control midgut cells appeared normal, with a clearly visible epithelial layer (EL), well distinguished gut lumen (GL), and normal peritrophic matrix (Pm). Midgut cells treated with NH-EDx and their major phytochemicals showed severe morphological alterations in the GL, EL, and Pm. The damage rate was particularly severe for NH-EDx as compared to its metabolites *n*-HDa and *n*-ODa. Correspondingly, the fourth instar larval midgut cells of *S. litura* were significantly affected after treatment with sublethal dosages of NH-EDx (Figure 11F), *n*-HDa (Figure 11G), and *n*-ODa (Figure 11H) as compared to the control (Figure 11E). Moreover, the brush border membrane (BBM), EL, and GL displayed the most significant cellular damage in NH-EDx, followed by *n*-HDa and *n*-ODa.

## 4. Discussion

It is marked that chemical-based insecticides/pesticides are having less impact against both mosquitoes and agriculture pests due to the upsurge in resistance of insect pests of different classes against commercial chemicals [42,43]. Synthetic insecticide resistance has been linked to the increased metabolism level in the insects, including the uplift of detoxifying enzymes or enzyme structural changes that increase the capacity of the metabolic rate [43]. The major factors that are responsible for insecticidal resistance include biological factors: (i) life cycle of the insect, (ii) population growth rate, (iii) genetics of the insect, and behavioral factors: (iv) movement and more importantly (v) exposure rate to chemicals [44]. In general, commercial chemicals contain artificial blends of two or three compounds that are quickly becoming resisted by pests due to their known mode of action [44]. To overcome this problem, natural-based insecticides chiefly derived from plant sources are widely evaluated for their insecticidal efficacy against different species of both mosquito vectors and agriculture pests [45,46,47,48,49].

Plant extracts and their major derivatives play a pivotal role in managing insect pests of both agricultural and medical importance due to their significant blends of natural chemicals including phenolic compounds, alkaloids, tannins, flavonoids, steroids, terpenes, coumarins, terpenoids, and lignins [50]. Also, the active ingredients contained in them are natural substances, not synthetic substances, with smooth degradation pathways in nature after application that result in less pollution to the environment. As botanical insecticides have many insecticidal ingredients with special modes of action, it is difficult for pests to develop pesticide resistance. Also, botanical insecticides generally have features of strong selectivity; low toxicity to humans, livestock, and natural enemies; and relatively low development and use costs [50]. Deadly diseases such as dengue spread by mosquito, affect >700 million people across the nation. The major vector responsible for spreading this disease is *A. aegypti*, which is widespread across all tropical and subtropical nations [23]. Correspondingly, *S. litura* is a crucial pest of agriculture, infesting more than 70% of plant crops globally and feeds on >290 plant species [4,51,52]. Both these pests are widespread across developing countries and threaten crops and human lives.

*E. divaricata* belongs to the family Compositae and is widely used in traditional medicine across South Asian countries. Moreover, *E. divaricata* contains a blend of natural compounds, including tannins, flavonoids, and steroids, with unique modes of action against bacterial and viral infections [36]. However, their insecticidal property remains uncharted. GC–MS analysis of NH-EDx showed nine major phytochemicals, with the highest peak area observed in *n*-HDa followed by *n*-ODa. Larvicidal screening of NH-EDx showed a prominent mortality rate in both the pests *S. litura* and *A. aegypti* at the maximum dosage of 1000 ppm. Moreover, the peak area compounds of NH-EDx also showed profound mortality rate against both insect pests. Previously, hexadecanoic acid ester derivatives showed a 70–98% decline in the oviposition rate in the groundnut pest *Caryedon serratus* at the maximum dosage of 1.0 mg L^−1^ [53]. Moreover, previous research proclaims that monoterpenes, alkanes, sesquiterpenes, fatty acids, and phenyl propanoids could demonstrate significant insecticidal and repellent activity against serious pests [54]. The bioactive metabolites of NH-EDx also showed high larvicidal activity against both pests. Previously, *Citrullus colocynthis* L. and its chief metabolite cucurbitacin E showed significant larvicidal activity (93.8%) against *S. litura* [4]. In parallel, bioactive andrographolide derived from *Andrographis paniculata* L. showed a prominent mortality rate against the dengue mosquito vector *A. aegypti* [55]. Comparably to the above results, the maximum lethal dosages of NH-EDx (1000 ppm), *n*-HDa (5 ppm), and *n*-ODa (5 ppm) delivered significant mortality rates against *A. aegypti* and *S. litura*.

Sublethal concentrations of NH-EDx (500 ppm), *n*-HDa (2.5 ppm), and *n*-ODa (2.5 ppm) showed substantial impacts in the growth and development of both *A. aegypti* and *S. litura*. The larval and pupal duration were significantly higher in the crude NH-EDx as compared to their bioactive metabolites across both the insect pests. Previously, Shaalan et al. [56] illustrated that the larval duration is blocked or altered when the larva is exposed to higher range of toxic chemicals. The above statement was well matched with our present results, as a significant extension of duration in larval and pupal stages was observed as compared to the control in both insect pests. In contrast, the adult longevity of both males and females of both the insect pests showed a significant reduction in a dose dependent manner, with the highest reductions in the maximum dosages of NH-EDx (500 ppm), *n*-HDa (2.5 ppm), and *n*-ODa (2.5 ppm). Adult longevity is considered as a restricted aspect for viral transmission in arthropods specifically, as the insect releases toxins that generally need a period of time to reach the midgut lining to begin replicate and spreads through the salivary glands to transmit virus [57].

The sublethal dosages of NH-EDx and their major metabolites showed significant alterations in the levels of midgut enzymes including α- and β-carboxylesterase and of detoxifying enzymes GST and CYP450 in both *A. aegypti* and *S. litura*. Previously, Koodalingam et al. [58] reported that the bioactive compounds derived from plant extracts contact to different insect species has displayed drift in the levels of carboxylesterase enzyme. The above statement was well supported with our present research, as the peak area compounds *n*-HDa and *n*-ODa displayed steady blockage in the esterase activity of both the pests. As dosage increased, α- and β-carboxylesterase levels decreased, and in contrast the detoxifying enzymes GST and CYP450 increased significantly. It is well known that GST and CYP450 are recognized as crucial biomarkers to detect resistance status in any insect pest [37,59]. The present results clearly display that NH-EDx decreased the enzyme ratio significantly as compared to the individual bioactive compounds *n*-HDa and *n*-ODa, and the level was slightly higher in *A. aegypti* as compared to *S. litura*.

Examination of midgut toxicity of NH-EDx and their chief derivatives displayed that the sublethal dosages caused severe damage to gut tissues such as the peritrophic matrix and also exhibited major changes in the alignment of the epithelial layer and brush border membrane as compared to the control. Previously, Ray et al. [60] clearly stated that the primary target of any plant metabolite is the midgut epithelium cells. It is well evident that the bioactive extracts of NH-EDx showed higher toxicity in the midgut profile of both the insect pests. Amala et al. [13] proclaimed that the peritrophic membrane is chiefly responsible for growth stimulus in the pests. The major metabolites derived from the hexane extracts of *Epaltes pygmaea* blocked the peritrophic membrane in the dengue vector and in turn decreased the development and growth rate of the insect pests.

## 5. Conclusions

The present investigation clearly displayed that NH-EDx and their bioactive metabolites *n*-HDa and *n*-ODa showed significant larvicidal, growth retardant, enzyme inhibition, and midgut toxicity effects against the crucial agricultural and medically challenging insect pests of ecological importance. Future perspectives will be focused on detecting the resistance and susceptibility of these bioactive compounds in the targeted pests as well as detecting the nontarget impacts of *n*-HDa and *n*-ODa against beneficial organisms sharing the same ecological niche of the targeted pest. Hopefully, the effective formulation of phytochemicals as commercial botanical insecticides will replace the current chemical pesticides, which are a global burden to our society, and which create chemical resistance in different insect pests.

## Figures and Tables

**Table 1:** Chemical characterization of NH-EDx (*n*-Hexane extracts of *E. divaricate*). R/T: retention time; PA: peak area.

Chemical Name	Formula	Weight (g/mol)	R/T (min)	PA (%)	Chemical Structure
Hexadecane	C_16_H_34_	226.4	9.033	0.75	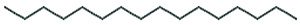
*n*-Eicosane	C_20_H_42_	282.5	13.23	1.13	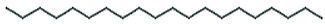
Tetratriacontane	C_34_H_70_	478.9	19.76	1.45	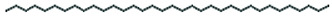
Ethyl-Hexadecanoate	C_18_H_36_O_2_	284.5	21.46	1.32	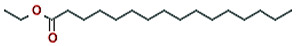
Neophytadiene	C_20_H_38_	278.5	21.97	0.83	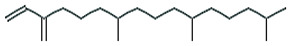
*n*-Hexatriacontane	C_36_H_74_	507	22.46	5.13	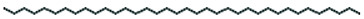
Hexacosane	C_26_H_54_	366.7	24.67	6.13	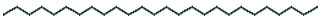
*n*-Octadecadienoic acid	**C_18_H_32_O_2_**	**280.4**	**31.78**	**14.63**	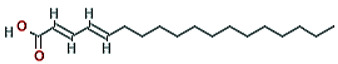
*n*-Hexadecanoic acid	**C_16_H_32_O_2_**	**256.4**	**33.45**	**6.73**	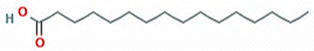

## Data Availability

Data is contained within the article.

## Sample Availability

Samples of the compounds are not available from the authors.

## References

[1] Senthil-Nathan S. (2013). Physiological and biochemical effect of Neem and other Meliaceae plants secondary metabolites against Lepidopteran insects. Front. Physiol..

[2] Murfadunnisa S., Vasantha-Srinivasan P., Ganesan R., Senthil-Nathan S., Kim T.J., Ponsankar A., Kumar S.D., Chandramohan D., Krutmuang P. (2019). Larvicidal and enzyme inhibition of essential oil from *Spheranthus amaranthroids* (Burm.) against lepidopteran pest *Spodoptera litura* (Fab.) and their impact on non-target earthworms. Biocatal. Agri. Biotechnol..

[3] Karthi S., Uthirarajan K., Manohar V., Venkatesan M., Chinnaperumal K., Vasantha-Srinivasan P., Krutmuang P. (2020). Larvicidal enzyme inhibition and repellent activity of red mangrove *Rhizophora mucronata* (Lam.) leaf extracts and their biomolecules against three medically challenging arthropod vectors. Molecules.

[4] Ponsakar A., Sahayaraj K., Senthil-Nathan S., Vasantha-Srinivasan P., Karthi S., Thanigaivel A., Petchidurai G., Madasamy M., Hunter W.B. (2020). Toxicity and developmental effect of cucurbitacin E from *Citrullus colocynthis* L. (Cucurbitales: Cucurbitaceae) against *Spodoptera litura* Fab. and a non-target earthworm *Eisenia fetida* Savigny. Environ. Sci. Pollut. Res..

[5] Chellappandian M., Senthil-Nathan S., Vasantha-Srinivasan P., Karthi S., Thanigaivel A., Kalaivani K., Sivanesh H., Stanley-Raja V., Chanthini K.M., Shyam-Sundar N. (2019). Target and non-target botanical pesticides effect of *Trichodesma indicum*(Linn) R. Br. and their chemical derivatives against the dengue vector, *Aedes aegypti* L.. Environ. Sci. Pollut. Res..

[6] Senthil-Nathan S., Choi M.Y., Paik C.H., Seo H.Y., Kalivani K., Kim J.D. (2008). Effect of azadirachtin on acetylcholinesterase (AChE) activity and histology of the brown plant hopper *Nilaparvata lugens* (Stal). Ecotoxicol. Environ. Saf..

[7] Thanigaivel A., Chandrasekaran R., Revathi K., Nisha S., Sathish-Narayanan S., Kirubakaran S.A., Senthil-Nathan S. (2012). Larvicidal efficacy of *Adhatoda vasica* (L.) Nees against the bancroftian filariasis vector *Culex quinquefasciatus* Say and dengue vector *Aedes aegypti* L. in in vitro condition. Parasitol. Res..

[8] Lija-Escaline J., Senthil-Nathan S., Thanigaivel A., Pradeepa V., Vasantha-Srinivasan P., Ponsankar A., Edwin E., Selin-Rani S., Abdel-Megeed A. (2015). Physiological and biochemical effects of chemical constituents from *Piper nigrum* Linn (Piperaceae) against the dengue vector *Aedes aegypti* Liston (Diptera: Culicidae). Parasitol. Res..

[9] Benelli G. (2016). Plant-mediated biosynthesis of nanoparticles as an emerging tool against mosquitoes of medical and veterinary importance: A review. Parasitol. Res..

[10] Senthil-Nathan S. (2015). A review of bio pesticides and their mode of action against insect pests. Environmental Sustainability- Role of Green Technologies.

[11] Selin-Rani S., Senthil-Nathan S., Thanigaivel A., Vasantha-Srinivasan P., Edwin E., Ponsankar A., Lija-Escaline J., Kalaivani K., Abdel-Megeed A., Hunter W.B. (2016). Toxicity and physiological effect of quercetin on generalist herbivore, *Spodoptera litura* Fab. and a non-target earthworm *Eisenia fetida* Savigny. Chemosphere.

[12] Senthil-Nathan S. (2020). A review of resistance mechanisms of synthetic insecticides and botanicals, phytochemicals, and essential oils as alternative larvicidal agents against mosquitoes. Front. Physiol..

[13] Amala K., Ganesan R., Karthi S., Senthil-Nathan S., Chellappandian M., Krutmunag P., Radhakrishnan R., Mohammad F., Ponsankar A., Vasantha-Srinivasan P. (2020). Larval and gut enzyme toxicity of n-hexane extract Epaltes pygmaea DC. against the arthropod vectors and its non-toxicity against aquatic predator. Toxin Rev..

[14] Huang S.H., Xian J.D., Kong S.Z., Li Y.C., Xie J.H., Lin J., Chen J.N., Wang H.F., Su Z.R. (2014). Insecticidal activity of pogostone against *Spodoptera litura* and *Spodoptera exigua* (Lepidoptera: Noctuidae). Pest. Manag. Sci..

[15] Selin-Rani S., Senthil-Nathan S., Revathi K., Chandrasekaran R., Thanigaivel A., Vasantha-Srinivasan P., Ponsankar A., Edwin E., Pradeepa V. (2016). Toxicity of *Alangium salvifolium* Wang chemical constituents against the tobacco cutworm *Spodoptera litura* Fab. Pest. Biochem. Physiol..

[16] Wheeler D.A., Isman M.B. (2001). Anti-feedant and toxic activity of *Trichilia americana* extract against the larvae of *Spodoptera litura*. Entomol. Exp. Appl..

[17] Koou S., Chong C., Vythilingam I., Lee C., Ng L. (2014). Insecticide resistance and its underlying mechanisms in field populations of *Aedes aegypti* adults (Diptera: Culicidae) in Singapore. Parasit. Vectors.

[18] Reegan A.D., Gandhi M.R., Paulraj M.G., Ignacimuthu S. (2015). Ovicidal and oviposition deterrent activities of medicinal plant extracts against *Aedes aegypti* L. and *Culex quinquefasciatus* Say mosquitoes (Diptera: Culicidae). Osong Public Health Res. Perspect..

[19] Thanigaivel A., Vasantha-Srinivasan P., Senthil-Nathan S., Edwin E., Ponsankar A., Chellappandian M., Selin-Rani S., Lija-Escaline J., Kalaivani K. (2016). Impact of *Terminalia chebula* Retz. against *Aedes aegypti* L. and non-target aquatic predatory insects. Ecotoxicol. Environ. Saf..

[20] Sivan A., Shriram A.N., Sunish I.P., Vidhya P.T. (2015). Studies on insecticide susceptibility of *Aedes aegypti* (Linn) and *Aedes albopictus* (Skuse) vectors of dengue and chikungunya in Andaman and Nicobar Islands, India. Parasitol. Res..

[21] Senthil-Nathan S., Kalaivani K. (2006). Combined effects of azadirachtin and nucleopolyhedrovirus (SpltNPV) on *Spodoptera litura* Fabricius (Lepidoptera: Noctuidae) larvae. Biol. Control..

[22] Senthil-Nathan S., Kalaivani K., Sehoon K. (2006). Effects of *Dysoxylum malabaricum* Bedd. (Meliaceae) extract on the malarial vector *Anopheles stephensi* Liston (Diptera: Culicidae). Bioresour. Technol..

[23] Yogarajalakshmi P., Poonguzhali T.N., Ganesan R., Karthi S., Senthil-Nathan S., Krutmuang P., Radhakrishnan N., Mohammad F., Kim T., Vasantha-Srinivasan S. (2020). Toxicological screening of marine red algae *Champia parvula* (C. Agardh) against the dengue mosquito vector *Aedes aegypti* (Linn.) and its non-toxicity against three beneficial aquatic predators. Aquat. Toxicol..

[24] Vasantha-Srinivasan P., Senthil-Nathan S., Ponsankar A., Thanigaivel A., Edwin E.S., Selin-Rani S., Chellappandian M., Pradeepa V., Lija-Escaline J., Kalaivani K. (2017). Comparative analysis of mosquito (Diptera: Culicidae: *Aedes aegypti* Liston) responses to the insecticide Temephos and plant derived essential oil derived from *Piper betle* L.. Ecotoxicol. Environ. Saf..

[25] Vasantha-Srinivasan P., Senthil-Nathan S., Thanigaivel A., Edwin E.S., Ponsankar A., Selin-Rani S., Pradeepa V., Sakthi-Bhagavathy M., Kalaivani K., Hunter W.B. (2016). Developmental response of Spodoptera litura Fab. to treatments of crude volatile oil from Piper betle L. and evaluation of toxicity to earthworm, Eudrilus eugeniae Kinb. Chemosphere.

[26] Edwin E., Vasantha-Srinivasan P., Senthil-Nathan S., Thanigaivel A., Ponsankar A., Selin-Rani S., Kalaivani K., Hunter W.B., Duraipandiyan V., Al-Dhabi N.A. (2016). Effect of andrographolide on phosphatases activity and cytotoxicity against Spodoptera litura. Invert. Surv. J..

[27] Annamalai M., Vasantha-Srinivasan P., Thanigaivel A., Chellappandian M., Karthi S., Mayabini J., Guru Pirasanna Pandi G., Totan A., Murugesan A.G., Senthil-Nathan S. (2017). Effect of thiamethoxam on growth, biomass of rice varieties and its specialized herbivore, *Scirpophaga incertulas* Walker. Physiol. Mol. Plant. Pathol..

[28] Dinesh-Kumar A., Srimaan E., Chellappandian M., Vasantha-Srinivasan P., Karthi S., Annamalai M., Thanigaivel A., Ponsankar A., Chanthini K., Shyam-Sundar N. (2018). Target and non-target response of *Swietenia Mahagoni* Jacq. chemical constituents against tobacco cutworm *Spodoptera litura* Fab. and earthworm, *Eudrilus eugeniae* Kinb. Chemosphere.

[29] Shin J., Vasantha-Srinivasan P., Kim K. (2018). The multi-faceted potential of plant-derived metabolites as antimicrobial agents against multidrug-resistant pathogens. Microbial pathog..

[30] Pradeepa V., Senthil-Nathan S., Sathish-Narayanan S., Selin-Rani S., Vasantha-Srinivasan P., Thanigaivel A., Ponsankar A., Edwin E.-S., Sakthi-Bagavathy M., Kalaivani K. (2016). Potential mode of action of a novel plumbagin as a mosquito repellent against the malarial vector Anopheles stephensi, (Culicidae: Diptera). Pesticide Biochem. Physiol..

[31] Senthil-Nathan S., Kalaivani K., Murugan K. (2005). Effects of neem limonoids on the malaria vector *Anopheles stephensi* Liston (Diptera: Culicidae). Acta Trop..

[32] Senthil-Nathan S., Chung P.G., Murugan K. (2005). Effect of bio-pesticides applied separately or together on Nutritional Indices of the Rice Leaffolder *Cnaphalocrocis medinalis*. Phytoparasitica.

[33] Hewawasam R.P., Jayatilaka K.A.P.W., Pathirana C., Muddduwa L.K.B. (2004). Hepatoprotective effect of *Epaltes divaricata* extract on carbon tetrachloride induced hepatotoxixity in mice. Ind. J. Med. Res..

[34] Amala K., Saraswathy A., Amerjothy S. (2013). GC-MS analysis of N-Hexane extract of *Epaltes divaricata* (L.) Cass. J. Pharmacog. Phytochem..

[35] Chah K.F., Eze C.A., Emuelosi C.E., Esimone C.O. (2006). Antibacterial and wound healing properties of methanolic extracts of some Nigerian medicinal plants. J. Ethnopharmacol..

[36] Glorybai L., Kannan B.K., Arasu M.V., Al-Dhabi N.A., Agastin P. (2015). Some biological activities of *Epaltes divaricata* L.—An in vitro study. Ann. Clin. Microbiol. Antimicrob..

[37] Vasantha-Srinivasan P., Chellappandian M., Senthil-Nathan S., Ponsankar A., Thanigaivel A., Karthi S., Edwin E., Selin-Rani S., Kalaivani K., Maggi F. (2018). A novel herbal product based on *Piper betle* and *Sphaeranthus indicus* essential oils: Toxicity, repellent activity and impact on detoxifying enzymes GST and CYP450 of *Aedes aegypti* Liston (Diptera: Culicidae). J. Asia-Pac. Entomol..

[38] World Health Organization (1981). Instruction for Determining the Susceptibility or Resistance of Mosquito Larvae to Insecticides.

[39] Abbott W.S. (1925). A method of computing the effectiveness of an insecticide. J. Econ. Entomol..

[40] Agra-Neto A.C., Napoleão T.H., Pontual E.V., Santos N.D.L., Luz L.A., Oliveira C.M.F., Melo-Santos M.A.V., Coelho L.C.B.B., Navarro D.M.A.F., Paiva P.M.G. (2015). Effect of *Moringa oleifera* lectins on survival and enzyme activities of *Aedes aegypti* larvae susceptible and resistant to organophosphate. Parasitol. Res..

[41] Thanigaivel A., Senthil-Nathan S., Vasantha-Srinivasan P., Edwin E., Ponsankar A., Selin-Rani S., Pradeepa V., Chellappandian M., Kalaivani K., Abdel-Megeed A. (2017). Chemicals isolated from *Justicia adhatoda* Linn reduce fitness of the mosquito. Arch. Insect Biochem. Physiol..

[42] Hemingway J., Ranson H. (2000). Insecticide resistance in insect vectors of human disease. Annu. Rev. Entomol..

[43] Silva W.J., Doria G.A.A., Maia R.T., Nunes R.S., Carvalho G.A., Blank A.F., Alves P.B., Marcal R.M., Cavalcanti S.C.H. (2008). Effects of essential oils on *Aedes aegypti* larvae: Alternatives to environmentally safe insecticides. Bioresour. Technol..

[44] Polson K.A., Brogdon W.G., Rawlins S.C., Chadee D.D. (2011). Characterization of insecticide resistance in Trinidadian strains of *Aedes aegypti* mosquitoes. Acta Trop..

[45] Piesik D., Wenda-Piesik A., Weaver D.K., Morrill W.L. (2007). Influence of Fusarium crown rot disease on semio-chemical production by wheat plants. J. phytopathol..

[46] Piesik D., Delaney K.J., Wenda-Piesik A., Sendel S., Tabaka P., Buszewski B. (2013). *Meligethes aeneus* pollen-feeding suppresses, and oviposition induces, *Brassica napus* volatiles: Beetle attraction/repellence to lilac aldehydes and veratrole. Chemoecology.

[47] Piesik D., Kalka I., Wenda-Piesik A., Bocianowski J. (2014). *Apion miniatum* Germ. herbivory on the mossy sorrel, *Rumex confertus* Willd.: Induced plant volatiles and weevil orientation responses. Pol. J. Environ. Stud..

[48] Vasantha-Srinivasan P., Karthi S., Ganesan R., Senthil-Nathan S., Krutmuang P., Chellappandian M., Radhakrishnan N., Ponsankar A., Karthick K., Nelofer A. (2021). The efficacy of methanolic extract of *Swietenia mahagoni* Jacq. (Meliaceae) and a commercial insecticide against laboratory and field strains of *Aedes aegypti* (Linn.) and their impact on its predator *Toxorhnchites splendens*. Biocatal. Agri. Biotechnol..

[49] Thanigaivel A., Chanthini M., Karthi S., Vasantha-Srinivasan P., Ponsakar A., Sivanesh H., Stanley-Raja V., Shyam-Sundar N., Narayanan K.R., Senthil-Nathan S. (2019). Toxic effect of essential oil and its compounds isolated from *Sphaeranthus amaranthoides* Burm. f. against dengue mosquito vector *Aedes aegypti* Linn. Pest. Biochem. Physiol..

[50] Chellappandian M., Vasantha-Srinivasan P., Senthil-Nathan S., Karthi S., Thanigaivel A., Ponsankar A., Kalaivani K., Hunter W.B. (2018). Botanical essential oils and uses as mosquitocides and repellents against dengue. Environ. Inter..

[51] Benelli G. (2018). Plant-borne compounds and nanoparticles: Challenges for medicine, parasitology and entomology. Environ. Sci. Pollut. Res..

[52] Zhao H.H., He J.T., Liu Z.X., Huang J.G. (2018). Cytotoxicity of chemical constituents from *Torricellia tiliifolia* DC. on *Spodoptera litura* (SL-1) cells. Pest. Biochem. Physiol..

[53] Tewari H., Jyothi K.N., Kasana V.K., Prasad A.R., Prasuna A.L. (2015). Insect attractant and oviposition enhancing activity of hexadecanoic acid ester derivatives for monitoring and trapping *Caryedon serratus*. J. Stored Prod. Res..

[54] Harborne J.B. (2003). Introduction to Ecological Biochemistry.

[55] Edwin E., Vasantha-Srinivasan P., Senthil-Nathan S., Thanigaivel A., Ponsankar A., Pradeepa V., Selin-Rani S., Kalaivani K., Hunter W.B., Abdel-Megeed A. (2016). Anti-dengue efficacy of bioactive andrographolide from Andrographis paniculata (Lamiales: Acanthaceae) against the primary dengue vector Aedes aegypti (Diptera: Culicidae). Acta Trop..

[56] Shaalan E.A.S., Canyon D.V., Younes M.W.F., Abdel-Wahab H., Mansour A. (2005). A review of botanical phytochemicals with mosquitocidal potential. Environ. Int..

[57] Hugo L.E., Jeffery J.A.L., Trewin B.J., Wockner L.F., Yen N.T., Le N.H., Nghia L.T., Hine E., Ryan P.A., Kay B.H. (2014). Adult survivorship of the dengue mosquito Aedes aegypti varies seasonally in Central Vietnam. PLoS Negl. Trop. Dis..

[58] Koodalingam A., Mullainadhan P., Arumugam M. (2011). Effects of extract of soapnut *Sapindus emarginatus* on esterases and phosphatases of the vector mosquito, *Aedes aegypti* (Diptera: Culicidae). Acta Trop..

[59] Zibaee A., Alborzi Z., Hoda H. (2014). A review on digestive TAG-lipases of insects. Arch. Phytopathol. Plant. Prot..

[60] Ray D., Pautou M.P., Meyran J.C. (1999). Histopathological effects of tannic acid on the midgut epithelium of some aquatic diptera larvae. J. Inver. Pathol..

